# Editorial: Food engineering technologies: Solutions to build sustainable and resilient food systems, and increase food and nutrition security

**DOI:** 10.3389/fnut.2022.1048332

**Published:** 2022-10-04

**Authors:** Emmanuel Purlis, Chiara Cevoli, Vanessa Jury

**Affiliations:** ^1^CIDCA, UNLP, Consejo Nacional de Investigaciones Científicas y Técnicas (CONICET), La Plata, Argentina; ^2^Department of Agricultural and Food Sciences, Alma Mater Studiorum, Università di Bologna, Cesena, Italy; ^3^Oniris, Université de Nantes, CNRS GEPEA, UMR 6144, Nantes, France

**Keywords:** resiliency, sustainability, nutrition security, food engineering, food systems

We are currently and we will continue to facing major global challenges concerning food and nutrition security. By 2050, food systems will need to supply safe, affordable and nutritious, socially and ethically accepted foods to ca. 10 billion people around the world, according to general estimations. Furthermore, this has to be done in a sustainable manner: we need to reduce and optimize the use of resources, e.g., food, energy, water, and land. In fact, many of the sustainability goals outlined by the United Nations are related to food systems.

Current food systems are highly integrated/globalized and present low resiliency, i.e., they are very susceptible to both local and global stresses such as economic, political, and natural disruptions and disasters. This lack of resiliency has been highlighted by the recent COVID-19 pandemic since beginning of 2020. For instance, the COVID-19 pandemic and adopted measures like lockdowns have triggered an increase of the number of people suffering food insecurity in developing countries, and a rise in food waste in developed countries. It is expected that similar stresses to food systems continue to appear in the (near) future, e.g., similar pandemics, economic shocks, and climate change effects at local and global scales. Therefore, it is of utmost importance to build resilient food systems to face future disruptions and reduce food and nutrition insecurity.

Solutions to these complex and dynamic problems are obviously not straightforward and require actions of multiple sectors, and a holistic approach. Regarding food systems, possible general solutions include:

- Design of more sustainable processes: less use of energy and water, and less/zero waste.
- Valorization of by-products, side-streams and “waste”.
- Increase the shelf life of foods.
- Improvement of nutritional profile of foods.
- Reduce the use of highly refined and dry ingredients, toward functional and sustainable ingredients.
- Novel and sustainable sources of raw materials, e.g., proteins, ingredients.
- Eco-design of shorter and resilient food supply chains.
- Development of robust local/regional food systems.

Considering this background, this Research Topic aims at disseminating *solutions based on food engineering technologies*, in order to build sustainable and resilient food systems, and increase food and nutrition security. Such solutions may involve:

- *Manufacturing solutions*: innovative and emerging non-thermal technologies, process intensification, shelf life extension, processing and valorization of by-products, side-streams and waste, use of regional raw materials, assessment of process sustainability.
- *Nutrition solutions*: nanoencapsulation, smart packaging, structuring, reduction of salt, sugar and saturated fats, use of less pure and more complex raw materials and ingredients, application of novel sources of proteins.
- *Supply chain solutions*: assessment of supply chain sustainability, systems engineering studies, management of concentrated, less refined and stable ingredients.

Contributions included in this collection cover a significant range of the mentioned solutions, providing examples of pertinent pathways to achieving the established goals.

Firstly, Sá et al. present a critical review about the advances and perspectives of emerging processing methods to improve plant protein quality and properties. In general, traditional thermal (intensive) processing is applied, but some disadvantages are high time- and energy-consuming operations, large water expenditure, and losses of desirable compounds in the final product. Instead, different emerging technologies have been investigated, e.g., ultrasound, microwave, supercritical fluids, pulsed electric field, high-pressure, ohmic heating, cold plasma, and enzymatic processes. These technologies can be applied alone or in combination for process intensification. Emerging methods are non-thermal or performed at mild temperatures/short times, thus providing a promising balance between the processing feasibility with reduced environmental impact and the increased nutritional aspects and techno-functionalities of the proteins. Authors highlight the broad perspective of these emerging technologies for achieving the adequate quality of plant-based proteins, although they indicate that further investigation is needed, especially regarding protein digestibility and amino acid composition of plant proteins when using such technologies. Finally, it is observed that industrial application feasibility requires additional development at more extensive scales than those implemented at the laboratory. A crucial question is whether plant proteins can be extracted by such methods at medium-large scale, while meeting nutritional and sensory requirements for human consumption.

Secondly, Cassani and Gomez-Zavaglia discuss in a review article about the current situation and opportunities of wastes arising from fruits and vegetables (availability, characterization) as potential sources of valuable ingredients (fiber, polyphenols, pigments), suitable to be incorporated into food, pharmaceutical and cosmeceutical products. Fruits and vegetables lead the ranking of food wastes and losses, e.g., 40–50% of their production; this current problem can be seen as an enormous opportunity to design strategic resilience plans for the recovery of valuable compounds in a circular economy framework. In particular, peels, pulps, pomaces and seed fractions of fruit and vegetables can be suitable raw materials for the recovery of different bioactive compounds, including fiber (pectin, prebiotic oligosaccharides), polyphenols and pigments. The authors also discuss about sustainable and feasible extraction/obtention technologies to valorize wastes, which depend on the particular compound. They highlight the need of translating efforts made at laboratory scale to industrial processes, in order to effectively take advantage of these cost-effective raw materials. On the other hand, it is claimed that valorization of fruits and vegetables by-products will not only contribute to environmentally sustainable practices but also to creation of dynamic and competitive regional economies, especially in developing countries and rural environments. Finally, authors analyze the benefits of implementing a circular economy strategy to develop supply chains, especially for companies, considering a comprehensive concept of sustainability, from environmental to financial and social aspects.

Thirdly, Ma et al. present a research article about the impact of ultrasound-assisted freezing on the flavor, microstructure, and myofibrillar proteins of large yellow croaker, an important marine cultured fish widely distributed in China. It is well-known that freezing is an efficient method to extend the shelf life of foods by controlling microbial growth and decreasing biochemical reactions, but quality of frozen fish depends on freezing method (i.e., freezing rate). Therefore, several innovative and emerging freezing techniques have been proposed to provide a promising solution to optimize the crystallization of frozen fish. In this study, the authors evaluated five different freezing treatments: air freezing; immersed freezing; ultrasound-assisted immersed freezing (UIF) linked with single frequency at 20 kHz (SUIF); UIF linked with dual frequency at 20/28 kHz (DUIF); and UIF linked with triple frequency at 20/28/40 kHz (TUIF). Results indicated that multifrequency ultrasonic treatment (TUIF) efficiently improved the flavor attributes and characteristics of myofibrillar proteins of large yellow croaker. The authors concluded that multifrequency ultrasound-assisted freezing can serve as an efficient way for improving food quality and nutritional profile in a sustainable way.

Fourthly, An et al. report the development of a sustainable film based on polylactic acid (PLA) with incorporated N-halamine compound (MC), as a promising antimicrobial food packaging material for fresh produce. N-halamines are a group of compounds containing one or more nitrogen-halogen covalent bond(s); this high-energy halide bond provides a strong oxidative state so that it is able to inactivate microorganisms effectively. Polylactic acids (PLA) has compostable properties and also comparable mechanical properties as polyethylene terephthalate (PET) and polystyrene (PS). Therefore, authors grafted MC onto PLA resins, in order to obtain an antimicrobial food packaging film with satisfactory mechanical strength and transparency. The developed PLA-MC films showed a high transparency, strong mechanical strength, thermal stability, water vapor barrier, and oxygen permeability property. Films of PLA with 0.25% MC inactivated seven logs (complete inactivation) of *S. aureus* and *E. coli* within 30 and 5 min of contact, respectively. In a pilot study, strawberries wrapped with MC incorporated films extended their shelf life to at least 5 days at room temperature. Authors concluded that due to the ease of fabrication and the effective biocidal property, these films have a wide range of potential applications in the field of food packaging to extend the shelf life of fresh produce.

To conclude, we expect that these contributions can inspire and encourage the development and application of food engineering-based solutions to build sustainable and resilient food systems, and increase food and nutrition security. We agree with contributing authors that more work is needed to evaluate at large scale the application feasibility of technological solutions developed at lab or pilot scale. Exploring, finding and effectively implementing the requested solutions require more than isolated efforts, i.e., it entails interaction and collaboration between both academic and industrial sectors.

## Author contributions

All authors listed have made a substantial, direct, and intellectual contribution to the work and approved it for publication.

## Conflict of interest

The authors declare that the research was conducted in the absence of any commercial or financial relationships that could be construed as a potential conflict of interest.

## Publisher's note

All claims expressed in this article are solely those of the authors and do not necessarily represent those of their affiliated organizations, or those of the publisher, the editors and the reviewers. Any product that may be evaluated in this article, or claim that may be made by its manufacturer, is not guaranteed or endorsed by the publisher.

